# *Toxocara cati* and Other Parasitic Enteropathogens: More Commonly Found in Owned Cats with Gastrointestinal Signs Than in Clinically Healthy Ones

**DOI:** 10.3390/pathogens10020198

**Published:** 2021-02-13

**Authors:** Aurora L. Ursache, Adriana Györke, Viorica Mircean, Mirabela O. Dumitrache, Andrei Răzvan Codea, Vasile Cozma

**Affiliations:** 1 Department of Parasitology and Parasitic Diseases, University of Agricultural Sciences and Veterinary Medicine Cluj-Napoca, 400372 Cluj-Napoca, Romania; auroralivia.ursache@gmail.com (A.L.U.); viorica.mircean@usamvcluj.ro (V.M.); mirabela.dumitrache@usamvcluj.ro (M.O.D.); vasile.cozma@usamvcluj.ro (V.C.); 2 Department of Internal Medicine, University of Agricultural Sciences and Veterinary Medicine Cluj-Napoca, 400372 Cluj-Napoca, Romania; razvan.codea@usamvcluj.ro; 3 Academy of Agriculture and Forestry Sciences Gheorghe Ionescu-Siseşti (A.S.A.S.), 011464 Bucharest, Romania

**Keywords:** *T. cati*, enteropathogens, parasites, gastrointestinal signs, cats, risk factors

## Abstract

Intestinal parasites are involved in the health and wellbeing of cats and some of them, due to their zoonotic potential, represent a problem for public health. This study aimed to assess the prevalence of parasitic infections in cats with gastrointestinal signs. Fecal samples collected from 137 cats were analyzed by the flotation method using a sodium chloride saturated solution. The overall prevalence of intestinal parasites was 50.4%. Intestinal parasites were significantly (*p* < 0.0001) more common in cats with digestive clinical signs (66.7%) than in clinically healthy ones (19.2%). *Toxocara cati* (40.2%) was the most frequently identified intestinal parasite, followed by *Cystoisospora* spp. (10.2%), *hookworms* (3.7%), *Taeniidae* (2.2%), *Giardia duodenalis* (2.2%), and *Toxoplasma gondii* (0.7%). *Toxocara cati* (53.3%, *p* < 0.0001) and *Cystoisospora* spp. (15.6%, *p* < 0.001) were significantly more frequently diagnosed in cats with clinical signs. A lack of deworming in the last three months (OR: 15.9), outdoor access (OR: 13.8), the presence of digestive symptoms (OR: 5.4), and young age (OR: 4.2) were identified as risk factors for *T. cati* infection by logistic regression analysis. Regardless of age, gastrointestinal signs like vomiting, diarrhea, and inappetence were positively associated with *T. cati*.

## 1. Introduction

In recent years, the number of cats increased considerably in urban areas worldwide. It is considered that the proven benefits of companion animals to the mental, emotional, and physical health of humans [1] might play an important role in this dynamic. By the year 2019, in Europe, approximately 106.4 million cats were estimated to be raised as pets and Romania is one of the countries with considerable interest in feline companionship, with 47% of households owning at least one cat [2,3]. Gastrointestinal disorders are a common concern of the owner when bringing the cat to veterinary clinics for a health check-up [4,5]. The identification of the underlying cause can be a challenge for clinicians, but it is desirable for an efficient and complete therapeutic protocol and management. Among dietary and stress-related problems or intestinal primary disorders, various pathogens such as viruses, bacteria, and parasites can be responsible for the development of gastrointestinal (GI) signs in companion animals [6,7]. Parasitic enteropathogens, both protozoa, and helminths, are numerous and one of the causes for gastrointestinal disturbances expressed by diarrhea, vomiting, and/or changes in appetite [8,9], therefore fecal testing must be taken into consideration for differential diagnosis whenever digestive clinical signs are present [10]. Moreover, it is necessary to protect human–animal interaction by limiting the exposure to various infectious agents that can represent a risk to both cats and humans, such as *Toxocara cati*, hookworms (*Ancylostoma tubaeforme/Uncinaria stenocephala*), *Giardia duodenalis)*, *Toxoplasma gondii*, *Dipylidium caninum,* and *Echinococcus multilocularis* [11,12].

*Toxocara cati* is a nematode frequently found in the intestine of domestic cats worldwide, with differences in prevalence based on geographical areas, laboratory methods used for diagnostics, and the structure of the tested feline population [13]. 

Cats can become infected by oral route with *T. cati* infective eggs from the environment, through the lactogenic transmission of larvae from the queen to the kittens, or by the consumption of paratenic hosts, such as mice or birds, that contain somatic larvae [14]. Patent infections contribute to environmental contamination through feces, as hundreds of thousands of *Toxocara* spp. non-embryonated eggs per animal are released daily [15]. 

In addition to the veterinary importance, *T. cati* represents a threat to public health, since it is able to cause human toxocariasis, a severe condition that has four major forms of development: visceral toxocariasis, neurotoxocariasis, ocular toxocariasis, and covert toxocariasis [16]. *Toxocara cati* is less incriminated than *T. canis*, but its zoonotic potential should not be underestimated [17]. Humans can acquire the infection by ingesting embryonated eggs from the environment and through the consumption of unwashed fruits or vegetables, or by ingesting infective larvae through consumption of raw meat [18,19]. *Toxocara cati* may be an important soil-transmitted helminth, especially in children. This can be positively influenced by the increased number of free-ranging cats and their defecating behavior near playgrounds, sandpits, and different urban outdoor public areas [20].

It is necessary to continuously assess the prevalence of zoonotic intestinal parasites such as *T. cati* in domestic cats along with the parasitic contamination of the environment, to obtain a realistic picture regarding the risk for animal and public health. In addition, epidemiological studies regarding the correlations between clinical signs and the involvement of enteropathogens such as *T. cati* are desirable in providing a better understanding of the medical aspects among feline populations for veterinarians. In this context, this study aimed to evaluate the prevalence of *T. cati* and other parasitic enteropathogens in cats with GI signs, but also to identify the risk factors associated with the parasitic infection.

## 2. Results

The age of the cats from this study was between 4 weeks and 15 years with an average of 26.03 (±3.68) months; 66 (48.2%) cats were under 6 months old (2.96 ± 0.19 months), 39 (28.5%) were between 6 months and 2 years old (14.64 ± 1.18 months), and 32 (23.4%) individuals were over 2 years old (77.23 ± 8.19 months). The ratio between males and females was 1.05. Most cats were mixed breed (128/137). Ninety-six (70.1%) out of 137 cats had outdoor access, and only 74 (54.0%) cats were dewormed in the last three months. More than half (65.7%) of the cats referred to our laboratory presented one or more digestive signs, such as vomiting (*n* = 21), diarrhea (*n* = 78), or inappetence (*n* = 31). Digestive signs were more common in outdoor cats (78.8%), kittens (63.3%), and in un-dewormed cats (53.3%).

Overall, 71 (51.84%) out of 137 examined fecal samples from the owned cats referred to our laboratory were positive for at least one parasitic pathogen, out of which 39.4% were single infections and 12.4% were mixed infections. A total number of sixty-nine cats were infected with intestinal parasites (50.4%). *Toxocara cati* (40.2%) was significantly (*x*^2^ = 131.14, *p* < 0.00001) the most frequently found intestinal parasite, followed by *Cystoisospora* spp. (10.2%), hookworms (3.7%), *Giardia duodenalis* (2.2%), *Taeniidae* (2.2%), and *Toxoplasma gondii*-like oocysts (0.7%) (Table 1). *Toxoplasma gondii*-like were identified as *T. gondii* oocysts by mouse bioassay and PCR [21]. Additionally, seven cats (5.1%) were positive for respiratory parasites namely *Aelurostrongylus abstrusus/Trochostrongylus brevior* (4.4%) and *Eucoleus aerophilus* (0.7%) (Table 1). 

The prevalence of intestinal parasites (overall, as single or mixed infection) was significantly higher (*p* < 0.05) in cats with GI signs than in cats without GI signs (Table 2). Additionally, *Cystoisospora* spp., *G. duodenalis,* and *T. gondii* were exclusively identified in cats with GI signs, whereas *T. cati* was predominantly identified in cats with digestive signs (Table 2). Nevertheless, 11 out of 12 mixed infections were registered in symptomatic cats, and *T. cati* was involved in all intestinal mixed infections. *Toxocara cati* and *Cystoiospora* spp. was the most commonly found co-infection (8.9%) and the only one significantly involved in cats with digestive signs [*X*^2^_(5,90)_ = 164.13, *p* ˂ 0.0001] (Table 2). This association was registered only in outdoor cats and mainly in kittens (7/8) (unshown data).

Respiratory parasites were identified in cats with digestive signs only in co-infection with other intestinal parasites as *T. cati, Cystoisospora* spp. and hookworms (Table 1). 

Forty-eight (53.3%) out of 90 cats with digestive symptoms were infected with *T. cati*, and the prevalence was significantly higher (*p* ˂ 0.001) in symptomatic cats compared with healthy ones (14.9%) (Table 2). According to the age category, 57.9% of kittens with digestive signs were infected with *T. cati*, 50.0% of young cats, and 36.4% of adult cats (Table 3). A decreasing trend of *T. cati* in symptomatic cats with age was observed, but this was not statistically significant [*x*^2^_(2,90)_ = 1.85, *p* = 0.40]. Indoor and dewormed cats with digestive signs were less commonly infected with *T. cati* and the prevalence was not significantly different from those without clinical signs (Table 3).

**Figure 1:**
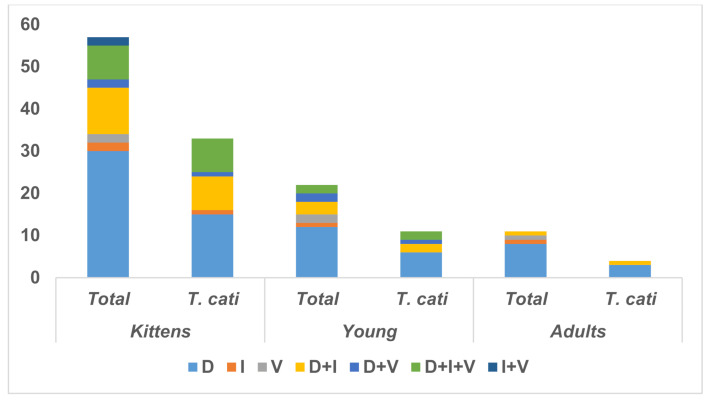
Digestive clinical signs identified in owned cats infected with *T. cati* by age group (D—diarrhea; I—inappetence; V—vomiting).

In kittens and young cats, diarrhea (47/48) was significantly [*x*^2^_(2,144)_ = 55.03, *p* < 0.00001] the most frequent digestive sign in cats infected with *T. cati* as a unique (24/48) sign or in combination (23/48) with other digestive signs (vomiting (12/48), and inappetence (22/48)) (Figure 1). 

Lack of deworming in the last three months (OR: 15.9), outdoor access (OR: 13.8), the presence of digestive signs (OR: 5.4), and young age (OR: 4.2) were identified as risk factors for *T. cati* infection in owned cats by logistic regression analysis (Table 4).

## 3. Discussion

Intestinal parasites are often diagnosed in companion animals; they affect the health and well-being of dogs and cats, and some of them are also of public health interest. Therefore, epidemiological studies are important in continuously updating data regarding the prevalence and associated risk factors for parasitic diseases. Regardless of the presence or absence of gastrointestinal signs, the overall prevalence of intestinal parasites in owned cats from this study was high (50.4%). In addition, we found respiratory nematodes with a lower prevalence in comparison with a previous study [22]. This is due to the poor sensitivity of the flotation method in comparison with Baermann or the molecular methods used for detecting the larvae of lungworms [22,23,24]. Two species of metastrongyloid lungworms were found in domestic cats in Romania [22], but no morphological or molecular differentiation between *Aelurostrongylus abstrusus* and *Troglostrongylus brevior* was performed in this study. Parasitic enteropathogens identified in this study in owned cats include two major zoonotic parasites: *T.* gondii and *T. cati.* Nevertheless, the potential risk for humans of the other three intestinal parasites should not be ignored (*G. duodenalis*, Taeniidae, hookworms). Neither the genetic assemblages of *G. duodenalis*, nor the differentiation of taeniid eggs between *Taenia* spp. and *Echinococcus granulosus* were performed. Therefore, the public threat of these parasites cannot be excluded. For public health purposes, future research focused on client-owned cats as a reservoir for both zoonotic enteropathogens and vector-borne pathogens are desirable, as recent studies highlight the exposure of cats to the latter [25]. *Toxocara cati* was the most prevalent intestinal parasite (40.2%), and significantly more prevalent in symptomatic (53.3%), than in asymptomatic owned cats (14.9%). Different studies have identified *T. cati* as the most frequently found parasite in domestic cats [13,26]. In Europe, *T. cati* prevalence varies between 7.2 and 83.3% with an average prevalence of 17.8% [11,26,27,28]. The prevalence of *T. cati* in household/owned and asymptomatic cats in Romania lies in the reported average for Europe according to this study and a previous one (20.3%) [29]. However, the prevalence of *T.cati* positive cats may differ depending on geographical regions, rural or urban areas, and exposure to risk factors. In western European countries, a lower prevalence is reported compared with southern, central, and eastern European countries [26,27].

Numerous studies aimed to establish the correlation between the exposure to different risk factors and infection with *T. cati* in cats. In the current study, outdoor access and a lack of deworming were identified as the main risk factors, as well as age and the presence of digestive clinical signs.

Cats with outdoor access were 13.82 times more susceptible to being *T. cati* positive than indoor cats. This emphasizes the possibility of free-roaming and predatory behavior significantly increasing the risk for infection also for cats with owners. This risk factor was also assessed by other authors [26,29,30] and a positive correlation was observed between the time a cat spends outdoors and the probability of a *T. cati* patent infection to occur [13]. The high percentage of cats with outdoor access (70.1) and an increased rate of infection with *T. cati* strongly suggests the important contribution this parasite may have in environmental contamination. Worldwide, the estimated prevalence of *Toxocara* spp. eggs in public places is 22% [31]. Differentiation between *T. canis* and *T.cati* eggs from soil samples is challenging and PCR techniques are recommended [32]. The low prevalence reported for *T.canis* in stool samples from the soil [33] can raise the question of whether or not *T. cati* represents a more significant parasitic contaminant, as observed in other studies [34,35]. Moreover, the presence of *T. cati* eggs in the hair of stray cats was assessed by others [36] and can represent an indicator of animal, human and environmental sources of contamination. Moreover, similar situations are likely to occur with free-ranging owned cats and should be further explored. Cats without an anthelmintic treatment in the last three months before sample collection were 15.9 more likely to be infected with *T. cati.* The prevalence of patent infection decreases if the frequency of anthelmintic treatment per year increases as shown in the study of Beugnet et al. [26]. Other studies did not find a positive association between patent *T. cati* infection and the time passed from the last deworming of the cats but suggest further investigations in this direction [13]. The European Scientific Council Companion Animal Parasites (ESCCAP) recommends a deworming frequency of four times a year and fecal examinations, but also a prevention and control management that can be adapted to individuals depending on exposure to risk factors. Kittens should start receiving deworming treatment every two weeks starting at three weeks of age until two weeks after weaning and monthly until they are six months. Moreover, for adult cats exposed to the infection through paratenic hosts from free-roaming and outside access, a monthly treatment is recommended [37]. 

Cats of all ages are prone to patent infections with *T. cati*, and therefore have the potential to be sources of contamination [38]. Nevertheless, in the current study, young age (0–2 years) represented a risk factor, with an increased rate of infection in kittens (51.5%). This is similar to the results obtain in other studies [13,29,39]. However, cats over 2 years should not be neglected as a potential reservoir for *T. cati* as the prevalence was still high (18.8%) probably due to the predatory behavior of outdoor cats (40%) [38]. *Toxocara cati* larvae that harbor in different tissues or organs of the paratenic hosts, such as small mammals or birds [40], once ingested by the definitive host, develop and reach adult stages in the small intestines [17]. Sex was not found to be a risk factor and the same results were obtained by others [11,41]. 

Multiparasitism has significant relevance for the course of the disease, and also provides information about which antiparasitic medication should be chosen according to the cat’s age and risks of exposure. *Toxocara cati* was found in all cases of mixed infections of intestinal pathogens (11/11), and the most prevalent mixed infection was between *T. cati* and *Cystoisospora* spp. in outdoor kittens.

Clinical signs are among of the main reasons behind owners’ concerns and therefore for veterinary consultation. Few studies have been made on the correlations between gastrointestinal signs and various pathogens in feline populations. Their results are of both epidemiological and clinical importance since they can provide the proper tools for veterinarians in establishing the most appropriate diagnostic and therapeutic protocols based on the risk factors and clinical aspects of each individual. In this study, the involvement of parasites, especially of *T. cati,* in cats with or without digestive symptoms was assessed. Parasitic enteropathogens were involved in a high percentage of cats with digestive symptoms (66.7%). The increased prevalence of parasites in cats with gastrointestinal signs was also found by others [42].

More than half of the cats (53.3%) that were brought with gastrointestinal signs were positive for *T. cati* infection. In contrast, in other studies, *Giardia* spp. was more frequently found in cats with diarrheic feces [6,43,44]. The rate of infection with *G. duodenalis* in cats is likely to be underestimated in this study, as the use of the flotation method has less sensitivity and a bigger probability of false-negative results in comparison with the more recommended assays such as PCR, ELISA, and immunochromatographic tests [45]. Therefore, an accurate frequency of feline giardiosis should be assessed, especially in diarrheic cats. However, similar findings to our study were reported in symptomatic owned cats from Italy [46] or in cats with diarrhea coming from different settlements (5–30%) in the USA [47]. 

In this study, the presence of digestive signs and associated risk factors for feline toxocarosis (young age, outdoor access, lack of deworming) strongly suggests infection with *T. cati* in cats. It was observed that regardless of the age of cats, diarrhea can be significantly linked to *T. cati* patent infection. In addition to the clinical and therapeutic implications of this correlation, it can be assumed that the zoonotic potential may be increased in cats with diarrhea. A high prevalence of toxocarosis was also seen in cats with inappetence, therefore, this clinical sign can also be suggestive for *T. cati*. Furthermore, the involvement of the parasite increased with the number of associated digestive signs. Although age can be a risk factor, the implication of *T. cati* as a pathogen involved in digestive signs was not influenced by the cat’s age. This emphasizes that the parasite should not be excluded from the list of differential diagnoses in gastrointestinal disturbances even for older cats.

An overall high percentage of parasitic pathogens was found in cats, but more importantly, in ones with digestive symptoms. Some of the identified parasites represent a concern in both animal and human health. Identifying the correlations between patent infections with *T. cati*, clinical manifestations and various associated factors in cats is of real interest for establishing the proper diagnosis and developing efficient measures for the treatment and control for feline infection, environmental contamination, and most importantly the zoonotic risk that this parasitic disease represents.

The results of this study confirm the high rate of infection with *T. cati* and the involvement of other parasitic enteropathogens in the etiology of gastrointestinal signs in cats. Fecal testing is highly recommended as a routine examination in clinically healthy animals and as an important diagnostic tool in individuals with digestive signs, which could also reduce environmental and human contamination.

## 4. Material and Methods

### 4.1. Animals, Samples, Questionnaires, and Samples Analysis 

Over three years, 137 cats that were presented at the Emergency and Critical Care Department of the Veterinary Medicine Faculty were tested microscopically for intestinal parasites at the Parasitology and Parasitic Diseases Department (University of Agricultural Sciences and Veterinary Medicine Cluj-Napoca—Faculty of Veterinary Medicine) for differential diagnosis or a periodical health check-up. Approximately 3–5 grams of feces per sample were analyzed using a flotation method in a hypersaturated sodium chloride solution (specific gravity 1.20) and examined after 30 minutes under a light microscope at a magnification of ×10, ×20, and ×40 when necessary. The identification of parasitic elements (cysts, oocysts, eggs) was based on morphological characteristics [48,49]. 

Positive samples for *T. gondii*-like oocysts were further analyzed by mouse bioassay and PCR techniques to differentiate between *T. gondii* and *Hammondia hammondi* oocysts and data were previously published [21].

Each cat owner received a questionnaire to fill in the identification data for cats. The information was later used to assess the possible risk factors that can influence the distribution of parasites: age, breed, and gender, the reason of presentation, digestive clinical signs, living conditions (indoor, outdoor), and deworming status in the last three months.

### 4.2. Statistical Analysis

Frequency, prevalence, and its 95% confidence interval were calculated for total infection, each parasite, co-infection, and the studied variables. The variables were represented by age, gender, living condition, the presence or absence of digestive signs, and deworming status. According to age, the cats were divided into three groups as follows: 0–6 months old (kittens), between 6 months and 2 years old (young cats), and over 2 years old (adult cats). A chi-square test was used to establish the differences in prevalence among studied variables. Risk factors associated with feline infection with *T. cati* and their odds ratio (OR) were analyzed by binary and multinomial logistic regression analysis, respectively. Statistical significance was established for a *p*-value equal to or less than 0.05. The statistical analysis was done with Epi Info^TM^ 7 software (CDC, Atlanta, GA, USA) [50].

## Figures and Tables

**Table 1:** Frequency (*n*), prevalence (%), and 95% confidence interval of internal parasites identified in the investigated cats.

	Frequency	Prevalence	95% Confidence Interval
Intestinal parasites	69	50.4	42.1–58.6
*T. cati*	55	40.2 ***	31.9–48.9
*Cystoisospora* spp.	14	10.2	5.7–16.6
*Giardia duodenalis*	3	2.2	0.5–6.3
*Hookworms*	5	3.7	1.2–8.3
*Taeniidae*	3	2.2	0.5–16.6
*Toxoplasma gondii*	1	0.7	0.0–4.0
Respiratory parasites	7	5.1	2.5–10.2
*Lungworms*	6	4.4	1.6–9.3
*Eucoleus aerophilus*	1	0.7	0.0–4.0
Total	71	51.8	43.5–60.0
Single infection	54	39.4	31.6–47.8
Mixed infection	17	12.4	7.4–19.1
*T.cati* + *Cystoisospora* spp.	8	5.8	2.6–11.2
*T.cati* + *Taeniidae*	2	1.5	0.2–5.2
*T.cati* + *lungworms*	2	1.5	0.2–5.2
*T. cati* + *G. duodenalis*	1	0.7	0.0–4.0
*T.cati* + *T. gondii*	1	0.7	0.0–4.0
*T. cati* + *E. aerophilus*	1	0.7	0.0–4.0
*Cystoisospora* spp. + lungworms	1	0.7	0.0–4.0
Hookworms + lungworms	1	0.7	0.0–4.8

Legend: hookworms—*Ancylostoma tubaeforme*/*Uncinaria stenocephala*; lungworms—*Aelurostrongylus abstrusus*/*Troglostrongylus* spp.; * *p* ˂ 0.05; ** *p* ˂ 0.01; *** *p* ˂ 0.001.

**Table 2:** Frequency, prevalence (*n* (%)), and 95% confidence interval of intestinal parasites identified in owned cats with and without digestive symptoms.

Parasite	Cats with Digestive Signs (*n* = 90)		Clinically Healthy Cats (*n* = 47)		*p*
	*n* (%)	95% CI	*n* (%)	95% CI	
*T. cati*	48 (53.3)	42.5–64.0	7 (14.9)	6.2–28.3	0.0000
*Cystoisospora* spp.	14 (15.6)	8.8–24.7	0	0.0–7.6	0.0009
*Giardia duodenalis*	3 (3.3)	0.7–9.4	0	0.0–7.6	0.1401
*Hookworms*	4 (4.4)	1.2–11.0	1 (2.1)	0.1–11.3	0.2783
*Taeniidae*	1 (1.1)	0.0–6.0	2 (4.3)	0.5–14.5	0.1547
*Toxoplasma gondii*	1 (1.1)	0.0–6.0	0	0.0–7.6	0.3284
Total intestinal parasites	60 (66.7)	56.4–75.6	9 (19.2)	10.4–32.5	<0.00001
Single infection	49 (54.4)	44.2–64.3	8 (17.0)	7.7–30.8	0.00003
Mixed infection	11 (12.2)	7.0–20.6	1 (2.1)	0.4–11.1	0.05
*T.cati* + *Cystoisospora* spp.	8 (8.9)	3.9–16.8	0	0.0–7.6	0.0155
*T.cati* + *Taeniidae*	1 (1.1)	0.0–6.0	1 (2.1)	0.1–11.3	0.3430
*T. cati* + *G. duodenalis*	1 (1.1)	0.0–6.0	0	0.0–7.6	0.3284
*T.cati* + *T. gondii*	1 (1.1)	0.0–6.0	0	0.0–7.6	0.3284

Legend: hookworms—*Ancylostoma tubaeforme*/*Uncinaria stenocephala*.

**Table 3:** Prevalence of *T. cati* by each variable in cats with and without digestive signs (diarrhea, vomiting, and inappetence).

	Total (*n* = 137)		Cats with Digestive Signs (*n* = 90)		Clinically Healthy Cats (*n* = 47)		*p*
	*n* (%)	95% CI	*n* (%)	95% CI	*n* (%)	95% CI	
**Age**							
<6 months	34 (51.5) **	38.9–64.0	33 (57.9)	44.1–70.9	1 (11.1)	0.3–48.3	0.006
6 months–2 years	15 (38.5)	23.4–55.4	11 (50.0)	28.2–71.8	4 (23.5)	6.8–49.9	0.05
>2 years	6 (18.8)	7.2–36.4	4 (36.4)	10.9–69.2	2 (9.5)	1.2–30.4	0.05
**Gender**							
Females	30 (47.8)	32.6–57.4	26 (65.0)	48.3–79.4	4 (14.8)	4.2–33.7	0.0000
Males	25 (35.7)	24.6–48.1	22 (44.0)	30.0–58.8	3 (15.0)	3.2–37.9	0.01
**Outdoor Access**							
Yes	53 (55.2) ***	44.7–65.4	46 (64.8)	52.5–75.8	7 (28.0)	12.1–49.4	0.0009
No	2 (4.9)	0.6–16.5	2 (10.5)	1.3–33.1	0	-	0.10
**Dewormed in the Last 3 Months**							
Yes	10 (13.5)	6.7–23.5	8 (19.1)	8.6–34.1	2 (6.3)	0.8–20.8	0.06
No	45 (71.4) ***	58.7–82.1	40 (83.3)	69.8–95.5	5 (33.3)	11.8–61.6	0.0002

Legend: * *p* < 0.05; ** *p* < 0.01; *** *p* < 0.001; CI—confidence interval.

**Table 4:** Risk factors for *T. cati* infection in domestic owned cats.

	OR	95% CI	*p*
**Age**			
0–24 months old (*n* = 105)	4.2	1.03–17.15	0.05
>24 months old (*n* = 32)	Ref.		
**Gender**			
Males (*n* = 70)	0.55	0.20–1.52	0.25
Females (*n* = 67)	Ref.		
**Outdoor Access**			
Yes (*n* = 96)	13.82	2.45–78.04	0.003
No (*n* = 41)	Ref.		
**Dewormed in the Last 3 Months**			
No (*n* = 74)	15.85	5.43–46.24	0.00001
Yes (*n* = 63)	Ref.		
**Digestive Clinical Signs**			
No (*n* = 47)	Ref.		
Yes (*n* = 90)	5.37	1.60–18.01	0.006

Legend: OR—odds ratio.

## Data Availability

Data sharing not applicable.

## References

[1] Barker S.B., Wolen A.R. (2008). The benefits of human–companion animal interaction: A review. J. Med. Vet. Educ..

[2] https://www.statista.com/statistics/516041/cat-population-europe-europe/.

[3] https://www.statista.com/statistics/515464/cat-ownership-european-union-eu-by-country/.

[4] Lund E.M., Armstrong P.J., Kirk C.A., Kolar L.M., Klausnor J.S. (1999). Health status and population characteristics of dogs and cats examined at private veterinary practices in the United States. J. Am. Vet. Med. Assoc..

[5] Jones P.H., Dawson S., Gaskell R.M., Coyne K.P., Tierney A., Setzkorn C., Radford A.D., Noble P.J. (2014). Surveillance of diarrhoea in small animal practice through the Small Animal Veterinary Surveillance Network (SAVSNET). Vet. J..

[6] Sabshin S.J., Levy J.K., Tupler T., Tucker S.J., Greiner E.C., Leutenegger C.M. (2012). Enteropathogens identified in cats entering a Florida animal shelter with normal feces or diarrhea. J. Am. Vet. Med. Assoc..

[7] Tello L., Perez-Freytes R. (2017). Fluid and electrolyte therapy during vomiting and diarrhea. Vet. Clin. N. Am. Small Anim. Pract..

[8] Deplazes P., Eckert J., Mathis A., Samson-Himmelstjerna G.V., Zahner H. (2016). Parasitology in Veterinary Medicine.

[9] Marks S.L., Washabau R.J., Day M.J. (2012). Chapter 11—Diarrhea. Canine and Feline Gastroenterology.

[10] Matz M.E., Guilford W.G. (2003). Laboratory procedures for the diagnosis of gastrointestinal tract diseases of dogs and cats. N. Z. Vet. J..

[11] Symeonidou I., Gelasakis A.I., Arsenopoulos K., Angelou A., Beugnet F., Papadopoulos E. (2018). Feline gastrointestinal parasitism in Greece: Emergent zoonotic species and associated risk factors. Parasites Vectors.

[12] Robertson I.D., Thompson R.C. (2002). Enteric parasitic zoonoses of domesticated dogs and cats. Microbes Infect..

[13] Nijsse R., Ploeger H.W., Wagenaar J.A., Mughini-Gras L. (2016). Prevalence and risk factors for patent *Toxocara* infections in cats and cat owners’ attitude towards deworming. Parasitol. Res..

[14] Overgaauw P.A., Nederland V. (1997). Aspects of *Toxocara* epidemiology: Toxocarosis in dogs and cats. Crit. Rev. Microbiol..

[15] Glickman L.T., Schantz P.M. (1981). Epidemiology and pathogenesis of zoonotic toxocariasis. Epidemiol. Rev..

[16] Macpherson C.N. (2013). The epidemiology and public health importance of toxocariasis: A zoonosis of global importance. Int. J. Parasitol..

[17] Fisher M. (2003). *Toxocara cati*: An underestimated zoonotic agent. Trends Parasitol..

[18] Glickman L.T., Shofer F.S. (1987). Zoonotic visceral and ocular larva migrans. Vet. Clin. N. Am. Small Anim. Pract..

[19] Taira K., Saeed I., Permin A., Kapel C.M.O. (2004). Zoonotic risk of *Toxocara canis* infection through consumption of pig or poultry viscera. Vet. Parasitol..

[20] Gerhold R.W., Jessup D.A. (2013). Zoonotic diseases associated with free-roaming cats. Zoonoses Public Health.

[21] Nedisan M.E., Ursache A.L., Dumitrache M.O., Mircean V., Cozma-Petruț A., Györke A. (2019). Intestinal toxoplasmosis in cats treated with Procox (case report). Sci. Parasitol..

[22] Györke A., Dumitrache M.O., Kalmár Z., Paştiu A.I., Mircean V. (2020). Molecular survey of metastrongyloid lungworms in domestic cats (*Felis silvestris catus*) from Romania: A retrospective study (2008–2011). Pathogens.

[23] Morelli S., Diakou A., Di Cesare A., Schnyder M., Colombo M., Strube C., Dimzas D., Latino R., Traversa D. (2020). Feline lungworms in Greece: Copromicroscopic, molecular and serological study. Parasitol. Res..

[24] Traversa D., Morelli S., Cassini R., Crisi P.E., Russi I., Grillotti E., Manzocchi S., Simonato G., Beraldo P., Viglietti A. (2019). Occurrence of canine and feline extra-intestinal nematodes in key endemic regions of Italy. Acta Trop..

[25] Morelli S., Crisi P.E., Di Cesare A., De Santis F., Barlaam A., Santoprete G., Parrinello C., Palermo S., Mancini P., Traversa D. (2019). Exposure of client-owned cats to zoonotic vector-borne pathogens: Clinic-pathological alterations and infection risk analysis. Comp. Immunol. Microbiol. Infect. Dis..

[26] Beugnet F., Bourdeau P., Chalvet-Monfray K., Cozma V., Farkas R., Guillot J., Halos L., Joachim A., Losson B., Miró G. (2014). Parasites of domestic owned cats in Europe: Co-infestations and risk factors. Parasites Vectors.

[27] Giannelli A., Capelli G., Joachim A., Hinney B., Losson B., Kirkova Z., René-Martellet M., Papadopoulos E., Farkas R., Napoli E. (2017). Lungworms and gastrointestinal parasites of domestic cats: A European perspective. Int. J. Parasitol..

[28] Rostami A., Sepidarkish M., Ma G., Wang T., Ebrahimi M., Fakhri Y., Mirjalali H., Hofmann A., Macpherson C.N., Hotez P.J. (2020). Global prevalence of *Toxocara* infection in cats. Adv. Parasitol..

[29] Mircean V., Titilincu A., Vasile C. (2010). Prevalence of endoparasites in household cat (*Felis catus*) populations from Transylvania (Romania) and association with risk factors. Vet. Parasitol..

[30] Näreaho A., Puomio J., Saarinen K., Jokelainen P., Juselius T., Sukura A. (2012). Feline intestinal parasites in Finland: Prevalence, risk factors and anthelmintic treatment practices. J. Feline Med. Surg..

[31] Fakhri Y., Gasser R.B., Rostami A., Fan C.K., Ghasemi S.M., Javanian M., Bayani M., Armoon B., Moradi B. (2018). Toxocara eggs in public places worldwide-A systematic review and meta-analysis. Environ. Pollut..

[32] Borecka A., Gawor J. (2008). Modification of gDNA extraction from soil for PCR designed for the routine examination of soil samples contaminated with *Toxocara* spp. eggs. J. Helminthol..

[33] Simonato G., Cassini R., Morelli S., Di Cesare A., La Torre F., Marcer F., Traversa D., Pietrobelli M., di Regalbono A.F. (2019). Contamination of Italian parks with canine helminth eggs and health risk perception of the public. Prev. Vet. Med..

[34] Otero D., Alho A.M., Nijsse R., Roelfsema J., Overgaauw P., de Carvalho L.M. (2018). Environmental contamination with *Toxocara* spp. eggs in public parks and playground sandpits of Greater Lisbon, Portugal. J. Infect. Public Health.

[35] Tyungu D.L., McCormick D., Lau C.L., Chang M., Murphy J.R., Hotez P.J., Mejia R., Pollack H. (2020). *Toxocara* species environmental contamination of public spaces in New York City. PLoS Negl. Trop. Dis..

[36] Bakhshani A., Maleki M., Haghparast A., Shirvan S.P., Borji H. (2019). A survey on *Toxocara* cati eggs on the hair of stray cats: A potential risk factor for human toxocariasis in Northeastern Iran. Comp. Immunol. Microbiol. Infect Dis..

[37] ESCCAP Worm Control in Dogs and Cats ESCCAP Guideline 01 Sixth Edition—February 2020. https://www.esccap.org/guidelines/gl1/.

[38] Overgaauw P.A., van Knapen F. (2013). Veterinary and public health aspects of *Toxocara* spp.. Vet. Parasitol..

[39] Barutzki D., Schaper R. (2003). Endoparasites in dogs and cats in Germany 1999–2002. Parasitol. Res..

[40] Strube C., Heuer L., Janecek E. (2013). *Toxocara* spp. infections in paratenic hosts. Vet. Parasitol..

[41] Zanzani S.A., Gazzonis A.L., Scarpa P., Berrilli F., Manfredi M.T. (2014). Intestinal parasites of owned dogs and cats from metropolitan and micropolitan areas: Prevalence, zoonotic risks, and pet owner awareness in northern Italy. BioMed Res. Int..

[42] López J., Abarca K., Paredes P., Inzunza E. (2006). Intestinal parasites in dogs and cats with gastrointestinal symptoms in Santiago, Chile. Rev. Med. Chile.

[43] López-Arias Á., Villar D., López-Osorio S., Calle-Vélez D., Chaparro-Gutiérrez J.J. (2019). *Giardia* is the most prevalent parasitic infection in dogs and cats with diarrhea in the city of Medellín, Colombia. Vet. Parasitol. Reg. Stud. Rep..

[44] Sauda F., Malandrucco L., De Liberato C., Perrucci S. (2019). Gastrointestinal parasites in shelter cats of central Italy. Vet. Parasitol. Reg. Stud. Rep..

[45] Symeonidou I., Gelasakis A.Ι., Miliotou A.N., Angelou A., Arsenopoulos K.V., Loukeri S., Papadopoulos E. (2020). Rapid on-site diagnosis of canine giardiosis: Time versus performance. Parasites Vectors.

[46] Riggio F., Mannella R., Ariti G., Perrucci S. (2013). Intestinal and lung parasites in owned dogs and cats from central Italy. Vet. Parasitol..

[47] Andersen L.A., Levy J.K., McManus C.M., McGorray S.P., Leutenegger C.M., Piccione J., Blackwelder L.K., Tucker S.J. (2018). Prevalence of enteropathogens in cats with and without diarrhea in four different management models for unowned cats in the southeast United States. Vet. J..

[48] Mircean V., Cozma V., Gyorke A. (2011). Coproscopical Diagnostic of Parasitic Diseases in Animals.

[49] Taylor M.A., Coop R.L., Wall R.L. Veterinary Parasitology.

[50] Dean A.G., Arner T.G., Sunki G.G., Friedman R., Lantinga M., Sangam S., Fagan R.F. (2011). Epi Info™, a Database and Statistics Program for Public Health Professionals.

